# Photoelectrochemical water splitting enhanced by self-assembled metal nanopillars embedded in an oxide semiconductor photoelectrode

**DOI:** 10.1038/ncomms11818

**Published:** 2016-06-03

**Authors:** Seiji Kawasaki, Ryota Takahashi, Takahisa Yamamoto, Masaki Kobayashi, Hiroshi Kumigashira, Jun Yoshinobu, Fumio Komori, Akihiko Kudo, Mikk Lippmaa

**Affiliations:** 1 Institute for Solid State Physics (ISSP), University of Tokyo, 5-1-5 Kashiwanoha, Kashiwa 277-8581, Japan; 2 Graduate School of Engineering, Nagoya University, Furo-cho, Chikusa-ku, Nagoya 464-8603, Japan; 3 Photon Factory, Institute of Materials Structure Science, High Energy Accelerator Research Organization (KEK), 1-1 Oho, Tsukuba 305-0801, Japan; 4 Department of Applied Chemistry, Faculty of Science, Tokyo University of Science, 1-3 Kagurazaka, Tokyo 162-8601, Japan; 5 Photocatalysis International Research Center, Research Institute for Science and Technology, Tokyo University of Science, 2641 Noda, Yamazaki 278-8510, Japan

## Abstract

Production of chemical fuels by direct solar energy conversion in a photoelectrochemical cell is of great practical interest for developing a sustainable energy system. Various nanoscale designs such as nanowires, nanotubes, heterostructures and nanocomposites have been explored to increase the energy conversion efficiency of photoelectrochemical water splitting. Here we demonstrate a self-organized nanocomposite material concept for enhancing the efficiency of photocarrier separation and electrochemical energy conversion. Mechanically robust photoelectrodes are formed by embedding self-assembled metal nanopillars in a semiconductor thin film, forming tubular Schottky junctions around each pillar. The photocarrier transport efficiency is strongly enhanced in the Schottky space charge regions while the pillars provide an efficient charge extraction path. Ir-doped SrTiO_3_ with embedded iridium metal nanopillars shows good operational stability in a water oxidation reaction and achieves over 80% utilization of photogenerated carriers under visible light in the 400- to 600-nm wavelength range.

## Abstract (web summary)

Nanoscale designs are known to increase the energy conversion efficiency of photoelectrochemical water splitting. Here, the authors report a self-organized nanocomposite formed by embedding self-assembled metal nanopillars in a semiconductor thin film, for enhanced photocarrier separation efficiency.

## Graphical abstract

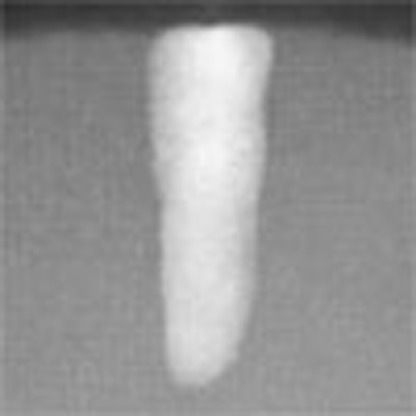

Potential benefits of direct hydrogen production by photoelectrochemical water splitting are well understood^1^, but the low energy conversion efficiency has so far prevented practical applications. The problem stems from a mismatch between the short photocarrier diffusion length and the large light absorption length in most water-stable semiconductors that are resistant to photocorrosion^2^. A common solution to this problem is to use various nanostructure designs that minimize the charge extraction path length and increase the photoelectrode surface area^3^,^4^,^5^. Spontaneous nanoscale phase separation is one possible technique of nanostructure synthesis that has been used for the development of various types of functional materials, including catalysts. For example, segregation of ferromagnetic CoPtCr grains in a CrO_*x*_ matrix has found use in high-performance perpendicular magnetic recording media^6^, spontaneous formation of ferromagnetic CoFe_2_O_4_ nanopillars in a ferroelectric BaTiO_3_ matrix leads to strong strain-mediated multiferroic coupling^7^, and efficient solar energy conversion has been achieved in organic solar cells containing a bicontinuous network of internal donor–acceptor heterojunctions^8^. Spontaneous segregation of pure metals is relatively rare in oxides, but has been reported recently for Fe, Co, Cu and several noble metals^9^,^10^,^11^,^12^.

Our interest is in developing a nanocomposite material combining an *n*-type oxide semiconductor matrix with embedded three-dimensional noble metal electrodes. We show that spontaneous noble metal segregation in a perovskite oxide thin film can be used in a simple single-step deposition process to form metal-oxide nanocomposites consisting of noble metal nanopillars in a semiconducting oxide matrix. The photocarrier transport efficiency is strongly enhanced in such nanocomposites due to the formation of Schottky space charge regions around the noble metal nanopillars while the metal pillars themselves provide an efficient charge extraction path to the photocatalyst film surface. Ir-doped SrTiO_3_ thin films with spontaneously formed embedded Ir metal nanopillars showed good operational stability in a water oxidation reaction and achieved over 80% internal quantum efficiency under visible light illumination in the 400- to 600-nm wavelength range.

## Results

### Photoelectrodes with embedded metal nanopillars

**Figure 1:**
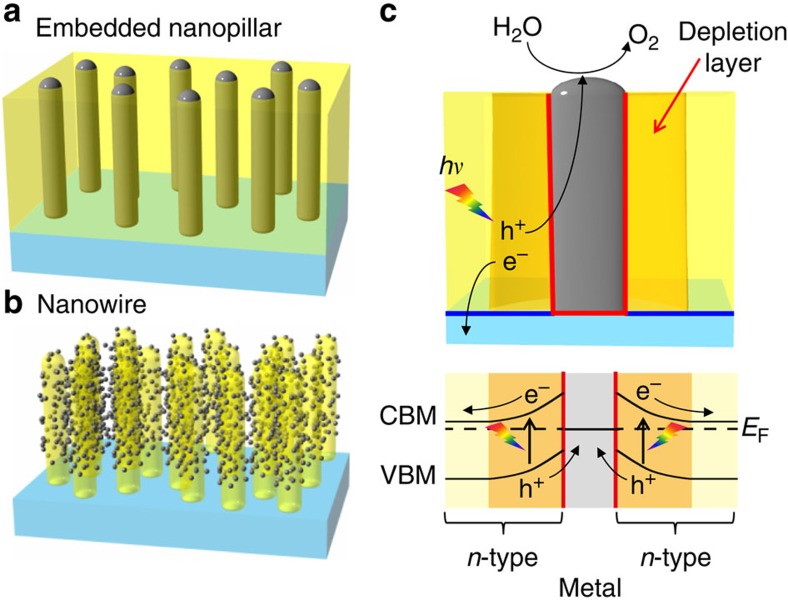
Nanocomposite semiconductor photoelectrode with embedded metal nanopillars for solar water splitting. (**a**) Schematic illustration of embedded metal nanopillars in a semiconductor film. (**b**) Schematic illustration of semiconductor nanowires coated with electrocatalyst nanoparticles. (**c**) Schematic illustration of a tubular three-dimensional Schottky junction formed around a metal nanopillar and a qualitative band structure diagram. Red lines mark Schottky barriers and blue lines mark Ohmic interfaces. CBM, conduction band minimum; VBM, the valence band maximum energy.

We use kinetic control of epitaxial thin film growth to impose a pillar-shaped structure on segregated metal inclusions, as illustrated in Fig. 1a. The metal pillars have two important roles in the photoelectrode. Since noble metals have high work functions, a Schottky barrier forms in an *n*-type oxide semiconductor in the vicinity of a metal pillar (Fig. 1c)^3^. The electric field in the Schottky barrier space-charge region provides the driving force for separating the photogenerated electrons from holes, reducing recombination losses. The nanopillar nanocomposite can thus efficiently separate photogenerated charges in a large volume fraction of a thin film by extending the space charge layer along the length of the nanopillar and effectively matching the charge generation depth with the optical absorption depth. The second important role of the metal nanopillars is to provide a conduction path for the generated charge to reach the liquid interface. If both the photoelectrode film and the substrate are *n*-type semiconductors, a Schottky barrier also forms at the interface between the metal nanopillar and the substrate, insulating the metal pillar from the substrate and driving the collected charge to the pillar–water interface. Similar design concepts have been explored before, either by embedding Pt nanopillars in an *n*-Si photoelectrode^13^ or by using an inverted geometry, where semiconductor nanowires are coated with electrocatalysts (Fig. 1b). The improvements of photoelectrochemical activity obtained with coated nanowires can be attributed to increased specific surface area and decreased charge transport distance, but the exposed nanowires are far less robust than embedded metal pillars inside a bulk semiconductor and require costly multistep fabrication procedures. The nanopillar composites explored in this work are all grown in a single deposition process, where suitable metal segregation is achieved by judicious choice of growth rate, temperature and oxygen pressure.

### Growth of self-assembled epitaxial metal nanopillars

Epitaxial nanopillar composite *M*(*x*%):SrTiO_3_ (*M*=Pt, Pd, Rh, Au and Ir) thin films were grown by pulsed laser deposition from polycrystalline SrTi_1−*x*_*M*_*x*_O_3_ (*x*=1, 3, 5 and 10%) ablation targets. In pulsed laser deposition growth, very high supersaturation of adatoms occurs on a microsecond scale when the ablation plume hits the film surface, followed by a long relaxation period of about a second. This leads to rapid nucleation and coalescence of noble metal clusters on the substrate surface during the growth of the first unit cell of the film. If the thermodynamic (temperature and oxygen pressure) and kinetic (growth rate) parameters are suitably balanced, the metal clusters remain stable on the surface and function as gettering sites for further metal adatoms delivered to the growth front, leading to the formation of vertical metal nanopillars instead of isolated nanoparticles, as happens in the case of homogeneous bulk segregation. Our experiments show that the segregation phenomenon is surprisingly common and occurs for several metals that bond weakly to oxygen, such as noble metals, which also happen to be efficient electrocatalysts for water splitting.

**Figure 2:**
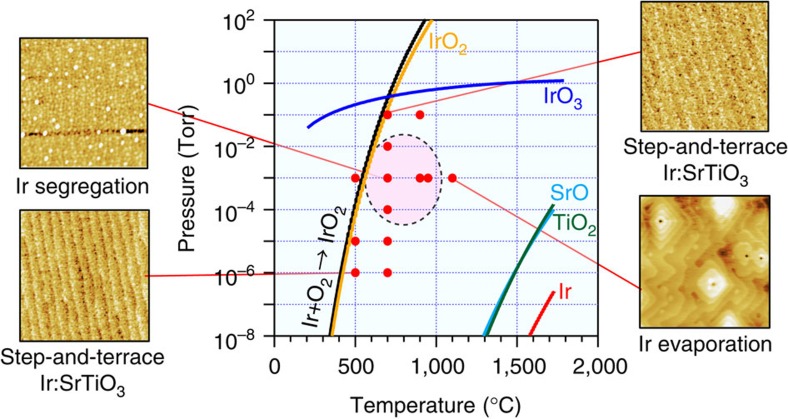
The effect of temperature and oxygen pressure on the formation of Ir nanopillars in SrTiO_3_. Deposition conditions of Ir(5%):SrTiO_3_ films (red circles) together with vapour pressure plots of binary oxides and Ir metal^15^,^16^. The black line marks the Ir Ellingham curve^17^. Ir metal nanopillars formed in the region encircled with the dotted line. The AFM imaging area was 1 × 1 μm^2^. The film thickness was 20 nm for all samples.

Our main focus is on Ir:SrTiO_3_ because it shows the highest photoelectrochemical activity under visible light irradiation among the metals that were tested. Intrinsic SrTiO_3_ with a bandgap of 3.2 eV can only absorb ultraviolet light and is insensitive to the visible part of the spectrum. Doping with Ir^4+^ ions at the Ti site leads to the formation of an occupied impurity level ∼1.1 eV above the O2*p* valence band maximum and an unoccupied state 0.8 eV below the conduction band minimum, effectively shrinking the bandgap. The Ir-doped films are dark yellow in colour and can utilize the visible part of the solar spectrum up to *λ*≈700 nm, *hv*>1.8 eV for photoelectrochemical reactions due to an optical transition from the occupied Ir^4+^ impurity level to the conduction band of SrTiO_3_ (ref. 14). Ir(5%):SrTiO_3_ films were grown on SrTiO_3_ (001) substrates at various temperatures and oxygen pressures. Deposition conditions of Ir(5%):SrTiO_3_ films are marked by red circles in Fig. 2, together with vapour pressure plots of binary oxides and Ir metal^15^,^16^, and the Ir Ellingham curve^17^. Ir metal segregation was found by atomic force microscopy (AFM) in films grown at around 700 °C and 10^−3^ Torr of oxygen at an ablation pulse rate of 2 Hz. At higher temperatures, above 1,100 °C, volatility of non-stoichiometric Ir oxides led to a loss of Ir from the films, while at lower temperatures, below 500 °C, Ir was incorporated in the SrTiO_3_ lattice without observable metal clustering. The growth window for the background oxygen pressure was also quite narrow. At high (10^−1^ Torr) or low (10^−6^ Torr) oxygen pressures, Ir metal segregation was not observed and clear step-and-terrace film surfaces were obtained. Under the optimum conditions of 700 °C and 10^−3^ Torr, an estimated 60% of Ir substituted at the Ti site of the SrTiO_3_ host material as Ir^4+^ and the remaining 40% segregated as pure Ir metal in nanopillars. The Ir metal segregation started at the initial film growth stage and was observable even in a 0.4-nm-thick single-unit cell film (Supplementary Figs 1 and 2), which means that the nanopillars nucleated directly on the substrate surface and grew continuously through the film. The diameter of the segregated metal pillars was uniform at ∼10 nm and independent of the Ir concentration in SrTiO_3_. Varying the Ir-doping level between 1 and 10% changed the areal density of the pillars without having a significant effect on the average pillar diameter (Supplementary Fig. 3).

### Structure analysis of nanopillar composite

**Figure 3:**
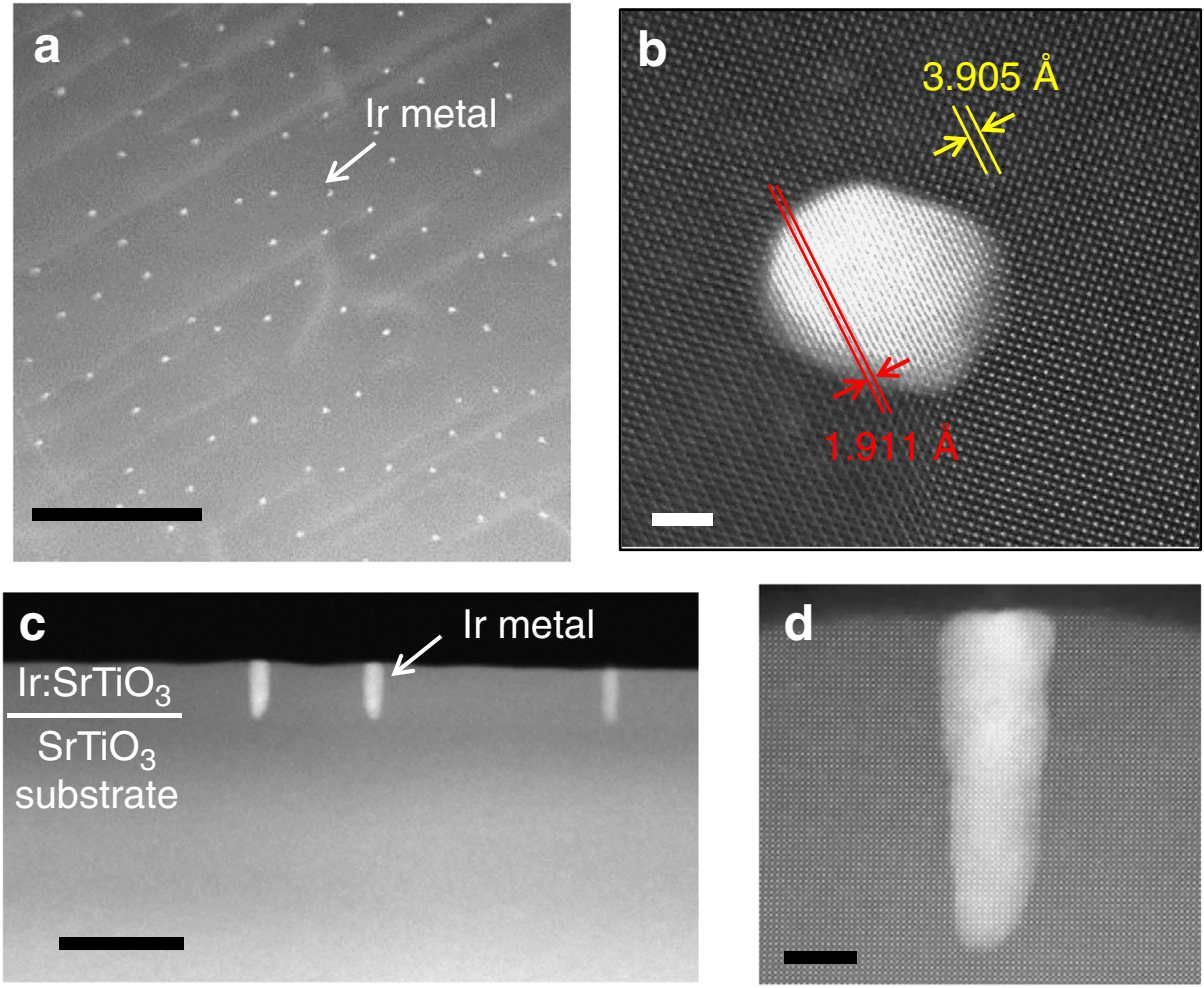
Structure of epitaxial Ir nanopillars. (**a**) Plan-view HAADF-STEM image of an Ir(5%):SrTiO_3_ (20 nm) film deposited at 700 °C, 10^−3^ Torr. The bright dots correspond to the Ir metal pillars. Scale bar, 200 nm. (**b**) A plan-view image of a single pillar shows that the Ir metal lattice is epitaxially matched with the SrTiO_3_ crystal lattice. The lattice parameters are 3.905 Å for SrTiO_3_ and 3.839 Å for the Ir metal. Scale bar, 2 nm. (**c**,**d**) Wide and narrow cross-section HAADF-STEM images of the same sample. Scale bars, 50 nm for **c** and 5 nm for **d**.

X-ray diffraction revealed that the nanopillars consisted of Ir metal with the bulk face-centred cubic structure and had a cube-on-cube epitaxial relationship with the SrTiO_3_ host lattice (Supplementary Figs 4 and 5). X-ray photoelectron spectroscopy showed that the nanopillar films contained Ir only in the Ir^4+^ and metallic states (Supplementary Fig. 6). The atomic-scale structure of the Ir nanopillars was analysed by high-angle annular dark-field scanning transmission electron microscopy (HAADF-STEM). The contrast of HAADF-STEM images is proportional to the atomic weight and the bright dots observed in the plan-view and cross-section images therefore correspond to the heavier Ir metal atoms (Fig. 3). Both in-plane and out-of-plane epitaxial relationships between the pillars and the SrTiO_3_ matrix were clearly visible in the HAADF-STEM images. The STEM images show that the crystal quality of an Ir:SrTiO_3_ film deposited at 700 °C and 10^−3^ Torr was exceptionally high, with the film lattice being indistinguishable from the SrTiO_3_ substrate lattice, as shown in Fig. 3c,d. This is quite unexpected for films grown at a relatively low temperature, considering that even homoepitaxial SrTiO_3_ films grown at 700 °C include many point defects induced by cation non-stoichiometry^18^. The lack of such defects in the film suggests that Ir metal segregation and rapid diffusion at the film surface suppressed defect formation in the bulk of the SrTiO_3_ film. The role of the Ir metal may thus be similar to a flux for SrTiO_3_ growth^19^, as indicated by the formation of ∼5 unit-cell-high SrTiO_3_ cones around the Ir nanopillars (Fig. 3c,d).

### Schottky junctions surrounding the metal nanopillars

**Figure 4:**
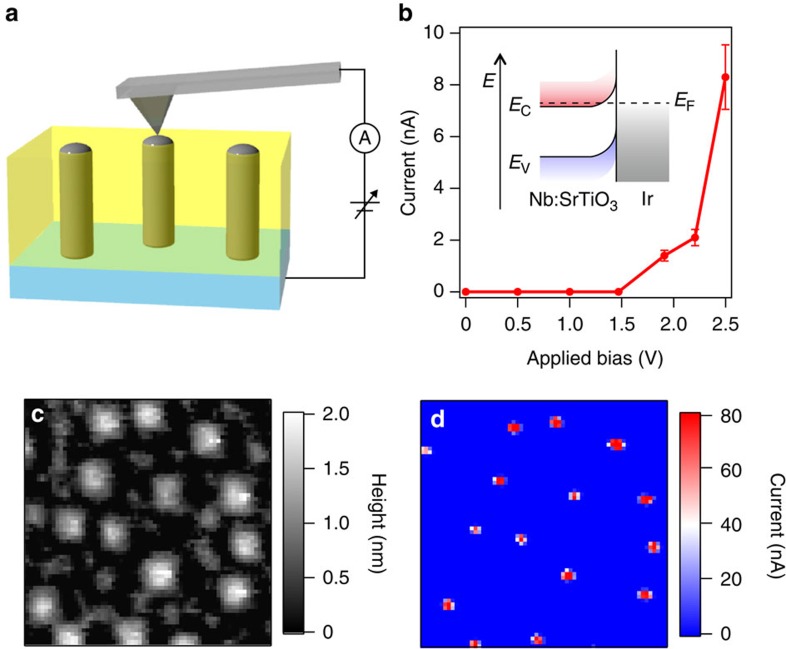
Local-probe conductivity analysis of a nanopillar composite film. (**a**) Schematic illustration of the current mapping measurement. (**b**) Current–voltage curve measured on a single Ir metal nanopillar. The inset illustrates the band diagram of the Schottky junction between an Ir pillar and the Nb(0.1%):SrTiO_3_ substrate. The error bars in **b** indicate the s. d. values of the current measured at a single nanopillar site. (**c**) Topographic AFM image (250 × 250 nm^2^) of an Ir(5%):SrTiO_3_ nanopillar film. (**d**) Current mapping obtained at an applied bias of 2.5 V, simultaneously acquired with **c**.

Since Ir metal has a very high work function of ∼5.7 eV (ref. 20) and SrTiO_3_ is an *n*-type semiconductor with a work function of 4.2–4.3 eV (ref. 21), a Schottky junction forms at the pillar interface with the Ir:SrTiO_3_ matrix. The formation of a depletion layer around the metal nanopillars increases the volume fraction of the film where strong internal electric fields enhance photocarrier separation (Fig. 1c). Local-probe transport analysis of the metal nanopillars with a conducting AFM tip (Fig. 4a) showed that the Ir nanopillars also formed a Schottky junction with the conducting Nb:SrTiO_3_ substrate (Fig. 4b). The built-in potential was ∼1.5 V, which is consistent with the work function difference between Ir metal and the Nb:SrTiO_3_ substrate. Figure 4c,d shows AFM topography and simultaneously acquired current mapping images of a Ir(5%):SrTiO_3_ film, measured at an applied ac bias of 2.5 V. Metallic conductivity at the pillar positions exceeded the Ir:SrTiO_3_ film conductivity by a factor of at least 10^5^. The strong upward band bending in the depletion region in the Ir:SrTiO_3_ film surrounding the Ir nanopillars enhances the spatial separation of photogenerated electron–hole pairs and the transport of photoexcited holes from the bulk to the surface, which plays an important role in achieving an energy-efficient photoelectrochemical water splitting reaction. Although the topmost surface of Ir may be oxidized, forming IrO_2_ at water interface, IrO_2_ is also an efficient electrocatalyst for water oxidation^22^.

### Photoelectrochemical properties of nanopillar composites

**Figure 5:**
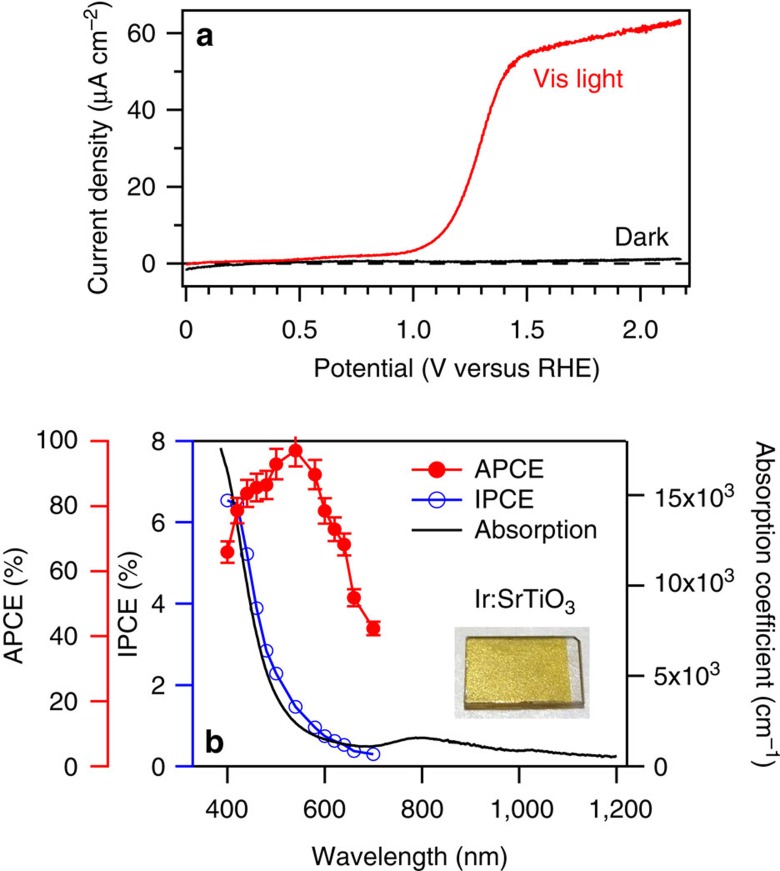
Photoelectrochemical properties of an Ir:SrTiO_3_ film containing Ir metal nanopillars. (**a**) Cyclic voltammetry scans of Ir(5%):SrTiO_3_ nanopillar film, measured with and without light irradiation (*λ*>420 nm, 70 mW cm^−2^). (**b**) absorbed photon-to-current efficienc (APCE) and incident photon-to-current efficiency (IPCE) measured at 1.6 V versus reversible hydrogen electrode (RHE), together with the light absorption coefficient plot. The film thickness was 20 nm, the estimated absorbance at 600 nm was 0.4%. The inset shows a photograph of an Ir(5%):SrTiO_3_ film deposited at 700 °C, 10^−3^ Torr on a nondoped SrTiO_3_(001) substrate. Electrolyte: 0.1 M KOH aqueous solution (pH=13.0); sweep rate: 20 mVs^−1^. The error bars in **b** assume that the main source of error is the 5% uncertainty in the film thickness.

The photoelectrochemical properties of the Ir(5%):SrTiO_3_ nanocomposite films were measured in a conventional three-electrode cell in a 0.1-M KOH aqueous solution (pH=13.0). Cyclic voltammetry curves under visible light irradiation (*λ*>420 nm) showed a strong anodic photocurrent (Fig. 5a), starting at 1.0 V versus a reversible hydrogen electrode. The sharp increase of the photocurrent is a clear indication that the photocarrier recombination rate is strongly suppressed in the bulk of the semiconductor photoelectrode. This can be attributed to the three-dimensional Schottky junctions surrounding the metal nanopillars. The optimum film thickness was ∼20 nm (Supplementary Fig. 7). The presence of the metal nanopillars was clearly detectable in Mott–Schottky plots (Supplementary Fig. 8). The photocurrent density did not decrease after 24-h operation of the photoelectrochemical cell, showing that the Ir nanopillars have good long-term operational stability (Supplementary Fig. 9). The total number of photocarriers flowing in 24 h (2.7 × 10^19^ cm^−2^) is much larger than the Ir concentration of 1.7 × 10^15^ cm^−2^ in a 20-nm-thick Ir(5%):SrTiO_3_ film. Therefore, the sample itself was very stable in alkaline conditions. The calculated absorbed photon-to-current efficiency and incident photon-to-current efficiency measured at 1.6 V versus reversible hydrogen electrode are shown in Fig. 5b together with the light absorption coefficient plot. The Ir:SrTiO_3_ films absorb visible light due to the Ir-induced impurity levels in the SrTiO_3_ bandgap^14^. As expected, the incident photon-to-current efficiency curve followed the light absorption curve, showing that the photocurrent is derived from the photocarrier generation in Ir:SrTiO_3_. The maximum absorbed photon-to-current efficiency value exceeded 80% in the 400- to 600-nm wavelength range.

## Discussion

The present work demonstrates that self-assembled nanopillar composite photoelectrodes can be grown easily in a single-step process and provide high internal quantum efficiency in the visible spectral range for water oxidation. The embedded metal nanopillars demonstrated in this work have major advantages over other nanostructure designs: automatic formation of Schottky depletion layers in the surrounding semiconductor films, a single-step fabrication process, mechanically robust film structure, and a photocorrosion-resistant surface. These factors simplify the synthesis process without compromising the operational longevity of a photoelectrode. The self-assembled nanocomposites studied here may find use not only in the efficient utilization of photocarriers but also in the development of various functional devices based on nanopillars and point contacts for spintronics, memories, multiferroic composites and photonic crystals.

## Methods

### Sample fabrication

SrTi_1−*x*_Ir_*x*_O_3_ powders were synthesized in a conventional solid state reaction and pressed into pellets for use as laser ablation targets^14^. All films were deposited on SrTiO_3_ (001) or conducting Nb(0.1%):SrTiO_3_ (001) substrates (Shinkosha) by pulsed laser deposition. A pulsed KrF excimer laser (Lambda Physik; COMPex201), operating at 248 nm, ∼1 J cm^−2^ and 2 Hz, was focused onto the target surface, creating a plasma plume that carried the evaporated material to the substrate surface. The oxygen gas pressure in the deposition chamber was adjusted with a variable leak valve from the chamber base pressure of about 5 × 10^−9^ to 1 Torr. The sample temperature was monitored with an optical pyrometer operating at 2 μm (Japan Sensor; FTC2) and focused onto the sample surface. The substrates were annealed at 1,000 °C and an oxygen pressure of 1 × 10^−5^ Torr for 10 min just before film depositions for cleaning the substrate surfaces. The film thickness was monitored by counting *in situ* reflection high-energy electron diffraction intensity oscillations with additional verification from the STEM images.

### Scanning transmission electron microscopy

Cross-sectional and plan views were taken in an aberration-corrected (CEOS GmbH) STEM (ARM-200F, JEOL Ltd.) at 200 kV. The probe-forming aperture semiangle was 22 mrad. The HAADF-STEM images were recorded with 81–228 mrad detectors. The cross-sectional TEM foils were prepared using a conventional method, including mechanical polishing and Ar ion milling (Model 691, Gatan Inc.). The plan-view TEM foils were prepared using a wedge-polishing method (MultiPrep system, ALLIED Inc.) and Ar ion milling.

### Scanning probe microscopy

Surface topography and current mapping analysis was done with a Shimadzu SPM-9600 AFM. Topography was measured in non-contact mode with Si cantilevers (Nanoworld NCHR), while conducting PtIr_5_-coated Si cantilevers (Nanoworld NCHPt) were used for current mapping in samples grown on Nb(0.1%):SrTiO_3_ (001) substrates. Measurements were done in a.c. mode with a lock-in amplifier (NF, LI5640). Variable signal amplitude at a fixed tip position was used for the *I*–*V* scans.

### Photoelectrochemical measurements

Film samples deposited on Nb(0.1%):SrTiO_3_ (001) substrates were attached to a home-made electrochemical cell made from a polytetrafluoroethylene block (Supplementary Fig. 10), where only the film surface was exposed to the electrolyte solution (0.1 M KOH aqueous solution, pH=13.0). Dissolved oxygen was removed by N_2_ bubbling. Ohmic back contact to the substrate was made with an InGa alloy. The typical area of a photoelectrode surface was 0.20 cm^2^. The measurements were performed in a three-electrode system using Pt (Nilaco, 99.98%) as a counter electrode and a Ag/AgCl 3.4 M KCl (eDAQ; ET072) miniature reference electrode. Cyclic voltammetry was measured both in dark conditions and under light irradiation from a 1-kW Xe-lamp (Ushio, UXL-1000D) with an L42 cutoff filter (*λ*>420 nm). The photon-to-current efficiency was evaluated using monochromatized light (full-width at half-maximum of 5 nm). The details are described in Supplementary Note 1. The light absorption coefficient of the Ir(5%):SrTiO_3_ film was measured with an Ultraviolet/Visible/Near-infrared spectrometer (Jasco V-570) for a 350-nm-thick film deposited at 700 °C and 1 × 10^−3^ Torr.

## Additional information

**How to cite this article:** Kawasaki, S. *et al*. Photoelectrochemical water splitting enhanced by self-assembled metal nanopillars embedded in an oxide semiconductor photoelectrode. *Nat. Commun.* 7:11818 doi: 10.1038/ncomms11818 (2016).

## Supplementary Material

Supplementary Information Supplementary Figures 1-10 and Supplementary Note 1
