# Supplementary material for: Photoelectrochemical water splitting enhanced by self-assembled metal nanopillars embedded in an oxide semiconductor photoelectrode
Source: Nat Commun. 2016 Jun 3;7:11818. doi: 10.1038/ncomms11818 (PMC4895796; doi:10.1038/ncomms11818)
Supplement: Supplementary Information — Supplementary Figures 1-10 and Supplementary Note 1 [file ncomms11818-s1.pdf]

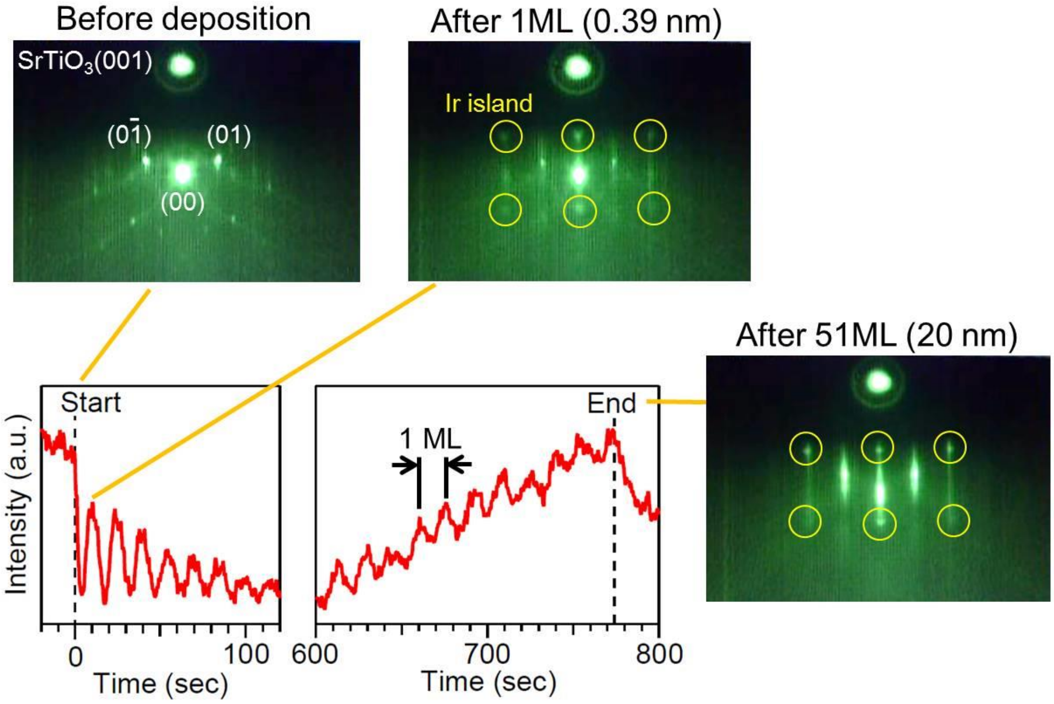

Supplementary Figure 1: RHEED observation during Ir nanopillar growth. Time dependence of RHEED specular peak intensity during the deposition of a Ir(5%):$SrTiO_3$ film on a $SrTiO_3$ (001) substrate at 700 °C, 10^{-3} Torr, together with typical RHEED patterns at the film thicknesses of 0, 1, and 51 ML. The images show that Ir metal started to segregate during the first monolayer of Ir:$SrTiO_3$ deposition, while Ir:$SrTiO_3$ film was grown in the layer-by-layer mode.

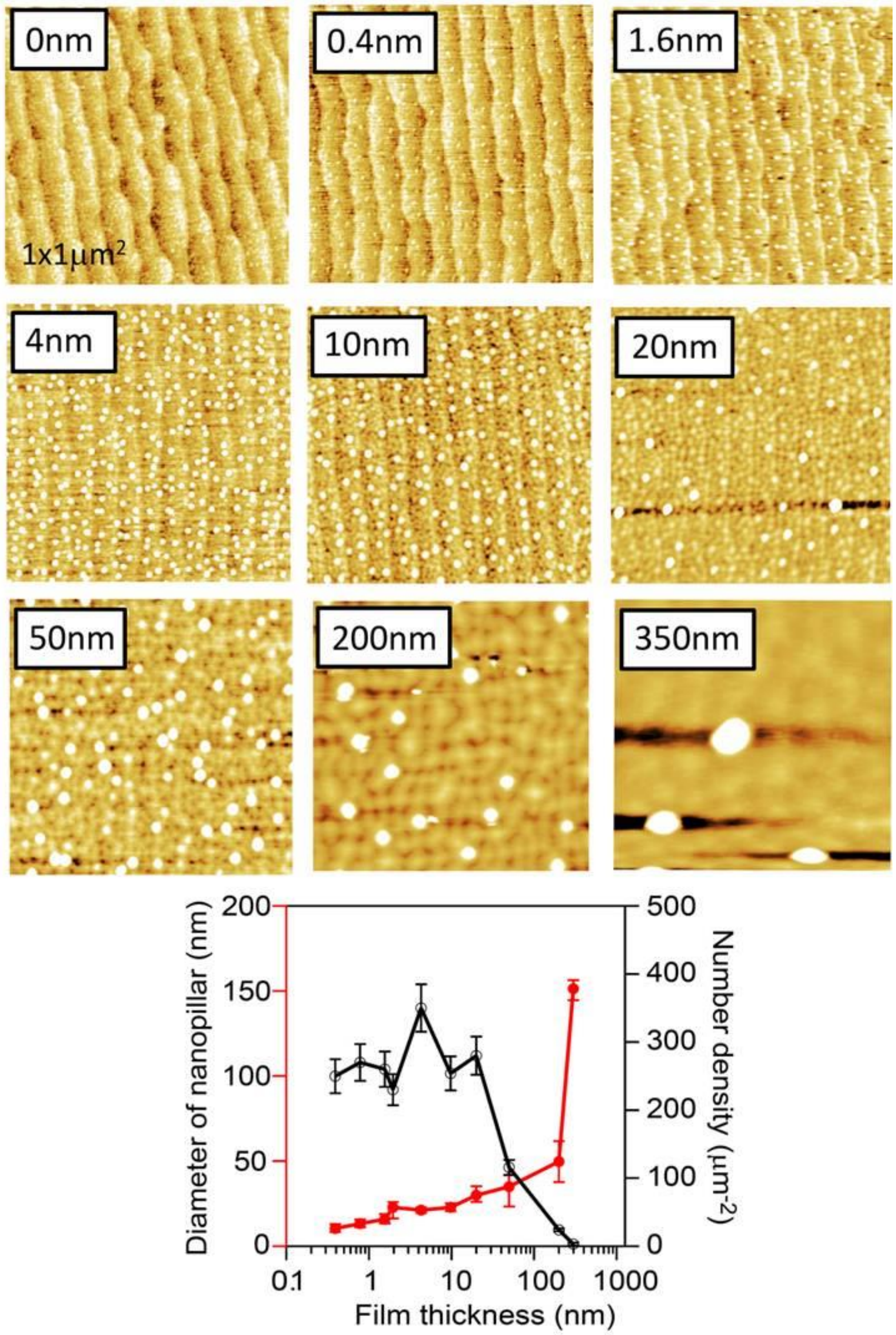

Supplementary Figure 2: Growth evolution of Ir metal nanopillars. AFM images (1×1 μm^2) of Ir(5%):$SrTiO_3$ films deposited at 700 °C, 10^{-3} Torr with different film thickness (0, 0.4, 1.6, 4, 10, 20, 50, 100, and 350 nm). The average diameter and the number density of nanopillars are summarized in a plot as a function of film thickness. Ir nanopillar nucleation occurs in the first unit cell layer. The AFM images indicated that the number density of the Ir nanopillars decreases rapidly after the film thickness exceeds 50 nm.

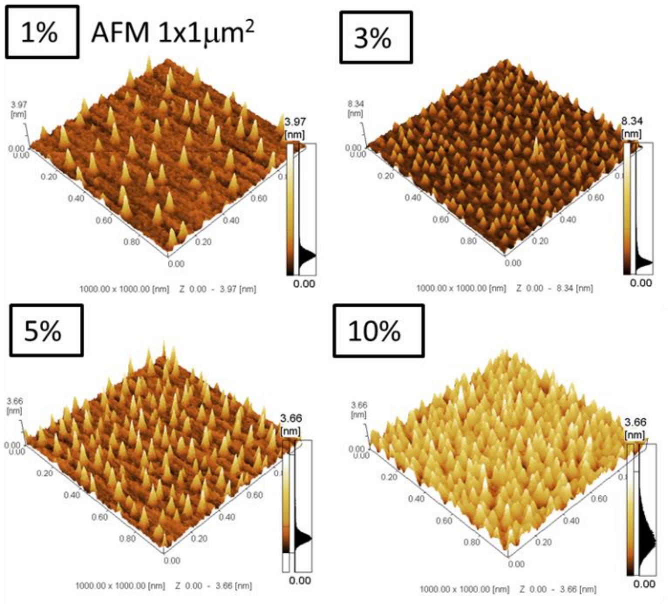

Supplementary Figure 3: Vaiation of nanopillar areal density with the Ir doping level. AFM images of $SrTi_{1-x}Ir_xO_3$(x=0.01, 0.03, 0.05, 0.10) films. The thickness was 20 nm for all films.

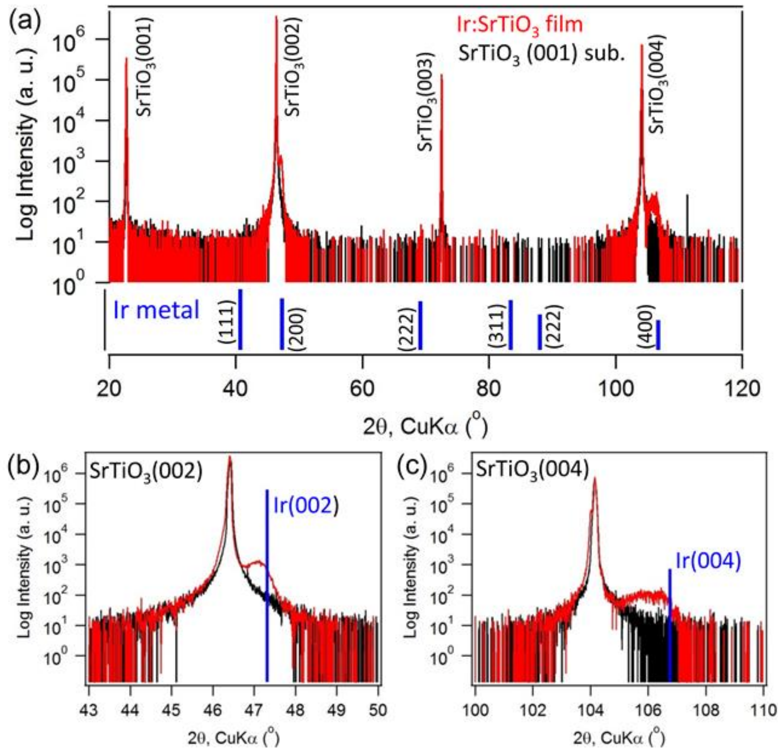

Supplementary Figure 4: Out-of-plane crystal orientation. a, wide ω-2θ scan for Ir(5%):$SrTiO_3$ film (350 nm) on $SrTiO_3$(001) and a bare $SrTiO_3$(001) substrate, together with reference peak positions for Ir metal (PDF card 03-065-1686). **b,c,** narrow scans near $SrTiO_3$(002) and (004) reflections.

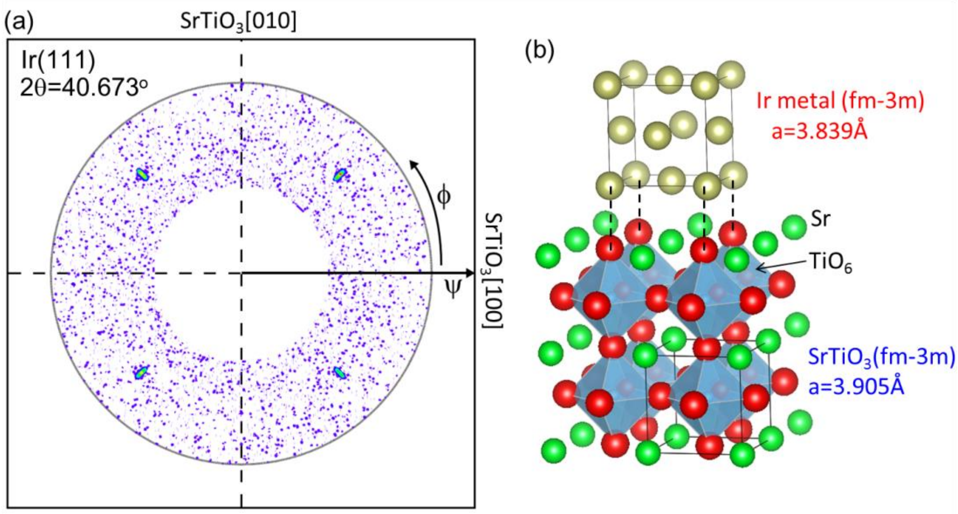

Supplementary Figure 5: In-plane orientation of the Ir nanopillars. a, pole figure of Ir(111) measured at 2θ=40.673° for Ir(5%):$SrTiO_3$ (350 nm) deposited at 700 °C, 10^{-3} Torr, clearly showing 4-fold symmetry of the Ir metal reflections. **b,** illustration of the cube-on-cube epitaxial relationship between the $SrTiO_3$(001) matrix and the Ir(001) metal nanopillars.

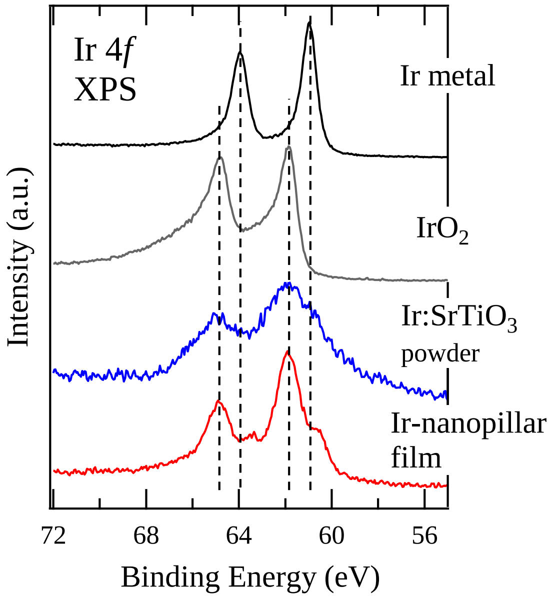

Supplementary Figure 6: Ir 4*f* photoelectron spectrum of an Ir nanopillar film. The Ir 4*f* spectra of Ir metal (Ir^0 reference), IrO_2 powder (Ir^{4+} reference), and fully oxidized Ir:$SrTiO_3$ powder (Ir^{4+} state) are also shown as references for the valence states. These spectra were taken with a monochromatized Al $K\alpha$ source ($h\nu$ = 1486.6 eV). The results show that the nanopillar film contains iridium in Ir^{4+} and metallic states. The binding energy was calibrated by the Fermi edge of an Au reference.

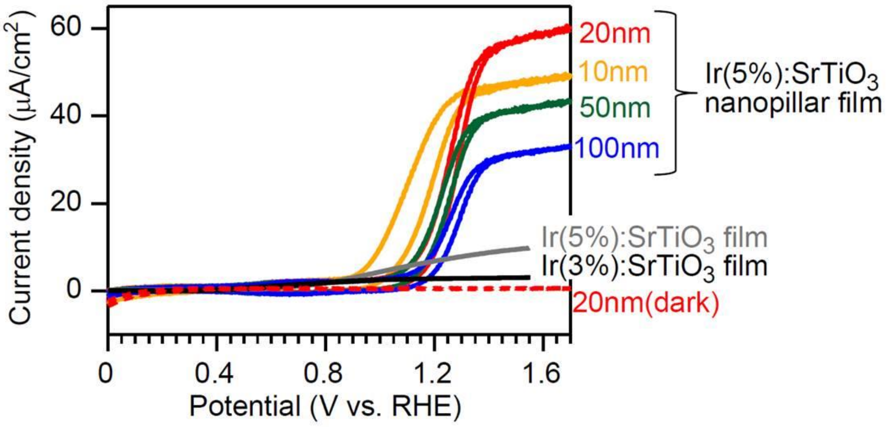

Supplementary Figure 7: Thickness dependence of photoelectrochemical activity of Ir nanopillar films. Cyclic voltammetry curves of Ir(5%):$SrTiO_3$ nanopillar films with different film thicknesses: 10, 20, 50, and 100 nm, showing that ~20 nm is the optimum film thickness. As references, cyclic voltammetry curves of 20-nm-thick Ir(3, 5%):$SrTiO_3$ flat epitaxial thin films are also displayed. Sweep rate: 20 mVs^{-1}, light source: 1 kW Xe-lamp a cut-off filter ($\lambda > 420$ nm, 70 mW/cm^2).

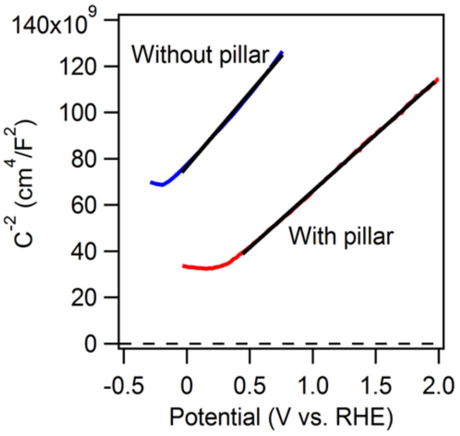

$$\frac{1}{C^2} = \frac{2}{e\varepsilon_0\varepsilon_r n}\left(U - U_{fb} - \frac{e}{kT}\right)$$

Ir-nanopillars	n (cm^{-3})	U_{fb} (V)
no	$7.4x10^{18}$	-1.20
yes	$9.6x10^{18}$	-0.35

Supplementary Figure 8: Mott-Schottky plot of Ir nanopillar film. Mott-Schottky plot of Ir(5%):$SrTiO_3$ (red line) with and (blue line) without Ir nanopillars. Black lines are conventional Mott-Schottky equation fitting curves. The obtained carrier density (n) and flat band potential (U_{fb}) are summarized in the table. The shift in the curves is due to a change of the reaction site from the $SrTiO_3$ surface to the Ir metal pillars. Film thickness: 20 nm, frequency: 100 Hz.

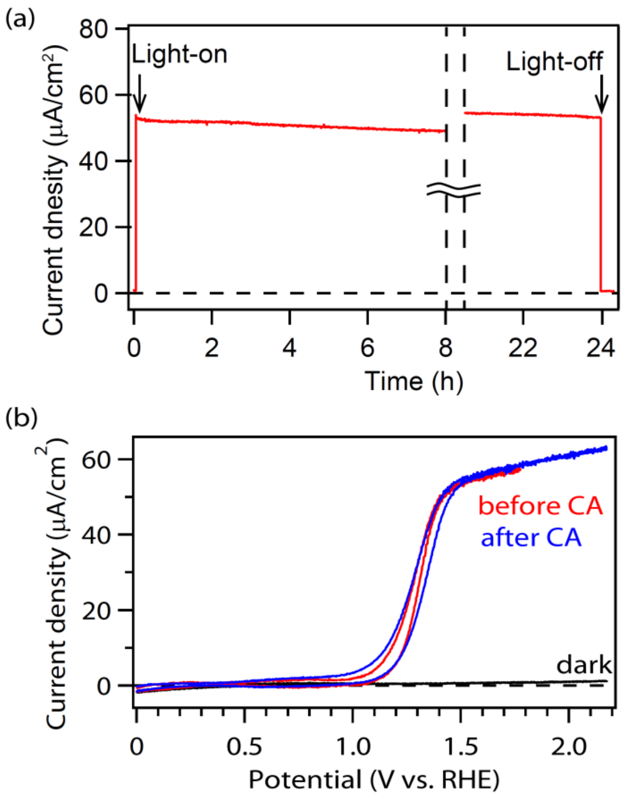

Supplementary Figure 9: Long-term operational stability. (a) Chronoamperometry (CA) of Ir(5%):$SrTiO_3$ with Ir nanopillars (the same sample as in Fig.5) measured at 1.6 V vs. RHE. (b) Cyclic voltammetry curves of Ir(5%):$SrTiO_3$ before and after the CA measurement. The results show that the photocurrent density did not deteriorate in 24 hours. Film thickness: 20 nm, electrolyte: 0.1 M KOH aqueous solution (pH = 13.0), sweep rate: 20 mVs^{-1}, light source: 1 kW Xe-lamp with a cut-off filter ($\lambda > 420$ nm, 70 mW/cm^2).

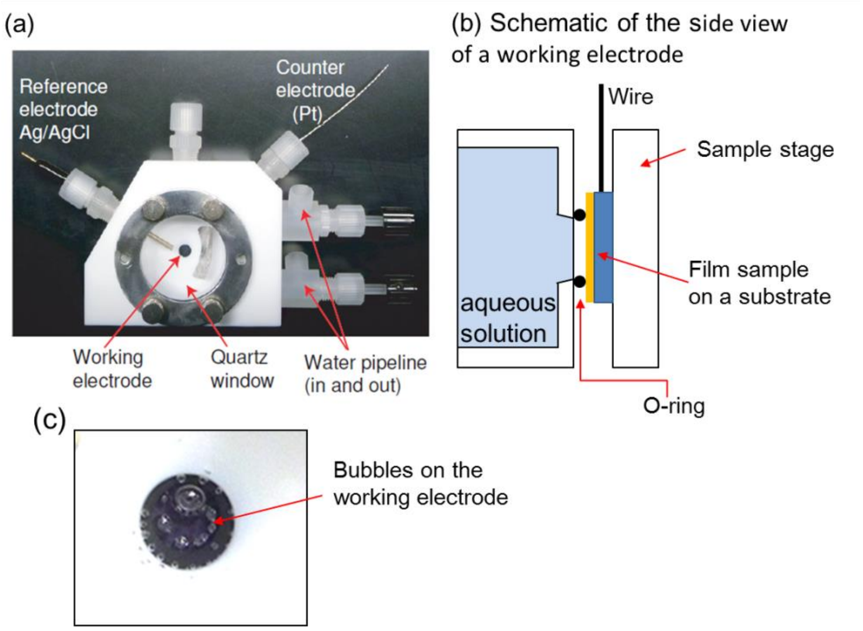

Supplementary Figure 10: Setup of photoelectrochemical measurements. (a) Photoelectrochemical cell used in our experiments. (b) A schematic of the side view of a working electrode attached to the cell. (c) An image showing oxygen bubbles on the working electrode surface of an Ir nanopillar sample at positive bias under visible light irradiation.

Supplementary Note 1

The incident photon-to-current efficiency (IPCE) and the absorbed photon-to-current efficiency (APCE) were calculated by the following equations:

$$IPCE(\%) = \frac{n_{photocarriers}}{n_{incident-photons}} \times 100 = \frac{1240\ j_{ph}(\lambda)}{P(\lambda)\ \lambda} \times 100 \quad (1)$$

$$APCE(\%) = \frac{n_{photocarriers}}{n_{adsorbed-photons}} \times 100 = \frac{1240\ j_{ph}(\lambda)}{P(\lambda)\ \lambda\ (1-10^{-\alpha(\lambda)\ d})} \times 100 \quad (2)$$

where $n_{photocarriers}$, $n_{incident-photons}$, $n_{adsorbed-photons}$ are the numbers of photocarriers, incident photons, and adsorbed photons, respectively. And $j_{ph}(\lambda)$, $P(\lambda)$, λ, $\alpha(\lambda)$, and d are photocurrent density, power density of incident light, wavelength of light, absorption coefficient, and the film thickness of a photoelectrode.
